# Salvage therapy with azacitidine for pediatric acute myeloid leukemia with t(16;21)(p11;q22)/*FUS‐ERG* and early relapse after allogeneic blood stem cell transplantation: A case report

**DOI:** 10.1002/ccr3.2461

**Published:** 2019-09-27

**Authors:** Dai Keino, Takashi Mori, Mizuho Morimoto, Kensuke Kondo, Tetsuya Mori, Akitoshi Kinoshita

**Affiliations:** ^1^ Department of Pediatrics St. Marianna University School of Medicine Kanagawa Japan

**Keywords:** acute myeloid leukemia, allogeneic hematopoietic stem cell transplantation, azacitidine, *FUS‐ERG*, salvage therapy, t(16;21)(p11;q22)

## Abstract

Acute myeloid leukemia (AML) with *FUS‐ERG* has a poor prognosis regardless of allo‐hematopoietic stem cell transplantation (HSCT). Maintenance therapy such as azacitidine after allo‐HSCT for AML with *FUS‐ERG* may be clinically meaningful.

## Graphical abstract

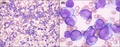

## 1 INTRODUCTION

We report a case of pediatric relapse AML with *FUS‐ERG* after allo‐hematopoietic stem cell transplantation who received salvage therapy with azacitidine (AZA). Our patient was able to achieve 2nd CR by AZA for approximately 8 months. We suggested that AZA may be a salvage therapy for AML with *FUS‐ERG*.

The t(16;21)(p11;q22) is a nonrandom chromosomal translocation that occurs in acute myeloid leukemia (AML), myelodysplastic syndrome that evolves into AML, blast crisis of chronic myelogenous leukemia, and rare cases of Ewing's tumors.^1^ This translocation leads to the production of the *FUS‐ERG* fusion transcript.^2^ The incidence of AML with *FUS‐ERG* is estimated to be 1% of all de novo and secondary cases of AML.^3^ AML with *FUS‐ERG* is reportedly associated with a poor prognosis and high rate of relapse.^4^, ^5^

AML with *FUS‐ERG* was defined as a high‐risk cytogenetic abnormality because it has been included in the high‐risk criteria in Japanese nationwide clinical trials for pediatric AML since 1999,^6^, ^7^ and the Japanese Pediatric Leukemia/Lymphoma Study Group (JPLSG) trial AML‐05 allocated *FUS‐ERG*‐positive AML to allogeneic hematopoietic stem cell transplantation (allo‐HSCT) as treatment for achieving the first complete remission (CR).^6^, ^8^

Here, we describe the clinical course of a patient who received salvage therapy with azacitidine (AZA) for pediatric AML with t(16;21)(p11;q22)/*FUS‐ERG* and experienced early relapse after the first CR with allo‐HSCT.

## 2 CASE REPORT

**Figure 1:**
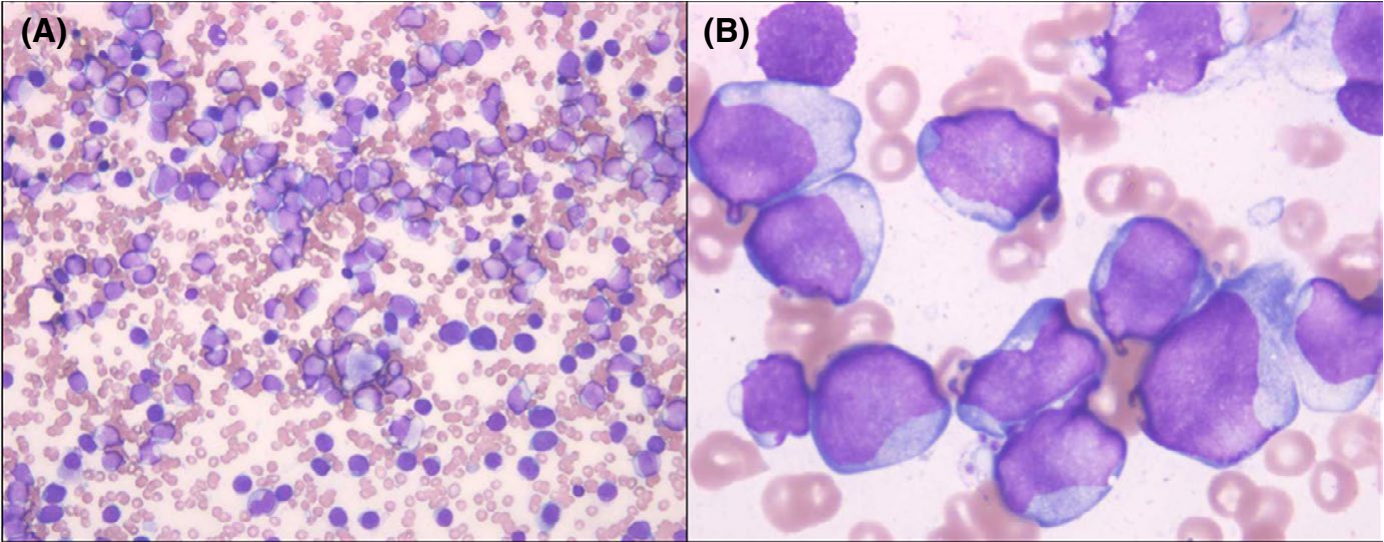
Bone marrow specimen (Wright‐Giemsa stain). A, ×400 and (B) ×1000: Leukemic cells are observed

A previously healthy 10‐year‐old boy was admitted to our hospital because of a fever. The physical examination showed petechial hemorrhage of the precordia and leg, but it did not indicate hepatosplenomegaly or lymphadenopathy. Results of the laboratory examination indicated leucocytosis, anemia, and thrombocytopenia. The following findings were observed: white blood cell count, 127,000/µL; neutrophil count, 2.0%; blast cell count, 94.5%; hemoglobin level, 9.3 g/dL; and platelet count, 1.7 × 10^4^/µL. Lactate dehydrogenase and transaminase levels were 2371 IU/L and within normal range, respectively. The bone marrow (BM) examination revealed hypercellular BM. The nucleated cell count was 26.4 × 10^4^/μL with a blast cell count of 71.8% (Figure 1A,B). The result of the peroxidase staining was negative, that of α‐naphthyl butyrate esterase staining was positive, that of naphthol AS‐D chloroacetate esterase staining was negative, and that of sodium fluoride staining was positive. As examined by flow cytometry, the immunophenotype of the blast cells showed positivity for cytoplasmic‐myeloperoxidase (42.9%), CD11b (75.1%), CD13 (95.9%), CD33 (99.2%), CD34 (98.0%), CD38 (35.9%), CD56 (96.2%), CD58 (70.6%), CD64 (43.1%), CD99 (93.4%), and CD117 (86.8%). Chromosomal banding of BM cells revealed 46,XY,del(6)(q21),t(16;21)(p11.2;q22),der(17)t(1;17)(q12;q25)[20]. The patient was negative for *FLT3‐ITD*, and the quantitative determination of *FUS‐ERG*‐messenger RNA (mRNA) in BM was 1,608,400 copies/μg RNA. The diagnosis of AML with t(16;21)(p11;q22)/*FUS‐ERG* and M5a according to the French‐American‐British classification was made based on the BM examination.

According to the AML‐05 protocol of the JPLSG,^8^ two courses of induction chemotherapy were administered, but CR was not achieved. The chemotherapy (granulocyte colony‐stimulating factor 5 μg/kg/d on days 1‐6, idarubicin 10 mg/m^2^/d on days 2‐4, fludarabine 30 mg/m^2^/d on days 2‐6, cytarabine 2 g/m^2^/d on days 2‐6) that included gemtuzumab ozogamicin (3 mg/m^2^/d on day 7) led to CR, which was followed by peripheral blood stem cell transplantation (PBSCT) from a human leukocyte antigen (HLA)‐matched sibling donor at 11 years of age. Sibling donor was sister. The conditioning regimen consisted of melphalan (L‐PAM) (90 mg/m^2^/d daily for 2 days) and buslfan (4 mg/kg/d daily for 4 days). Graft‐versus‐host disease (GVHD) prophylaxis was performed with short‐course methotrexate (MTX) and cyclosporine. BM engraftment was achieved 12 days after PBSCT, but veno‐occlusive disease (VOD) and infective endocarditis developed 20 and 68 days after PBSCT, respectively. Although GVHD prophylaxis had been discontinued because of VOD, he did not have acute GVHD.

At 145 days, the BM examination revealed the presence of blast cells (3%), 46,XY,del(6)(q21),t(16;21)(p11.2;q22),der(17)t(1;17)(q12;q25)[1]/46,XX[19] in the chromosome banding, and an increased expression of *WT1*‐mRNA (8,000 copies/μg RNA). He was diagnosed as having a cytogenetic relapse after PBSCT. At 156 days, AZA was administered (100 mg/m^2^/d for 5 days, every 28 days).^9^ After two courses of AZA were administered, CR was achieved a second time and chromosomal banding of BM cells revealed 46,XX[16]. The quantitative determination of *WT1*‐mRNA in peripheral blood was significantly reduced to 140 copies/μg RNA after three courses of AZA. For approximately 8 months, eight courses of AZA were administered, but he was diagnosed as having relapse in the BM and central nervous system (CNS) a second time. The hematotoxicity of AZA was neutropenia of Grades III‐IV and febrile neutropenia.

Although low‐dose Ara‐C and aclarubicin with concomitant use of a granulocyte colony‐stimulating factor regimen and triple intrathecal therapy (TIT) consisted of cytarabine (30 mg), methotrexate (12 mg), and hydrocortisone (25 mg) were administered, a third CR was not achieved. TIT was performed a total of 7×, and cerebrospinal fluid test revealed that the leukemia cells were negative. He underwent cranial irradiation (14 Gy) and subsequently received a reduced‐intensity conditioning regimen consisting of fludarabine (30 mg/m^2^/d for 4 days), cytarabine (2 g/m^2^/d for 4 days), L‐PAM (60 mg/m^2^/d for 3 days), and low‐dose total body irradiation (4 Gy) with HLA‐matched cord blood transplantation (CBT). GVHD prophylaxis was performed with short‐course methotrexate (MTX) and tacrolimus. Thrombotic microangiopathy developed 9 days after CBT, and primary graft failure (GF) was diagnosed 20 days after CBT. Therefore, he underwent PBSCT with an HLA‐matched sibling donor without conditioning regimen 20 days after CBT because of GF.

Although BM engraftment was achieved, he died of septic shock due to *Klebsiella pneumoniae* at approximately 3 months after CBT. According to the autopsy results, the presence of leukemic cells was not confirmed.

## 3 DISCUSSION

The CD56 antigen is expressed in natural killer/T‐cell lymphoma, multiple myeloma, and AML. Expression of the CD56 antigen in AML with *FUS‐ERG* was reported to be associated with failure of CR, early relapse, and an unfavorable prognosis.^3^ CD56 was highly expressed in our patient's leukemic cells. Further, he did not achieve CR after the administration of two courses of induction chemotherapy according to the AML‐05 protocol, and he experienced early recurrence after PBSCT.

Noort et al reported that no difference in the incidence of relapse could be observed between the MRD‐positive and MRD‐negative AML patients with *FUS‐ERG*.^5^ And the outcomes of allo‐HSCT for AML with *FUS‐ERG* have been reported in Japan.^10^ Despite all patients receiving allo‐HSCT, 12 achieved their first CR, and 12 of 14 patients eventually died (nine died of AML relapse and three died of transplant‐related toxicities). This fact indicates that allo‐HSCT is not a curative option for AML with *FUS‐ERG* and that a novel therapeutic approach is needed to improve patients’ outcomes. Considering that AZA was effective in our patient, epigenetic drugs may become a novel therapy option.

Low‐dose AZA maintenance therapy after allo‐HSCT has been reported in patients with AML and myelodysplastic syndrome.^11^, ^12^ AZA after allo‐HSCT can induce a CD8^+^ T‐cell response to tumor antigens, raising the possibility that it may have the potential to augment a graft‐versus‐leukemia (GVL) response.^13^

It has been reported that a high expression of *ERG* is linked with an unfavorable prognosis in a subgroup of leukemic patients with AML and acute T‐lymphoblastic leukemia.^14^ It has also been reported that several accompanying mutation in epigenetic regulators (*DNMT3A*, *ASXL1*, *BCOR*) by targeted NGS approach in AML with *FUS‐ERG* cases.^15^ *ERG*‐positive prostate cancer cells have been reported to induce epigenetic activation of Tudor domain‐containing protein 1, and histone deacetylase inhibitors induce apoptosis and affect the acetylation status of p53.^16^, ^17^

We administered AZA for pediatric AML with *FUS‐ERG* that early relapse occurred after the first CR with allo‐HSCT. The patient was able to maintain CR a second time for approximately 8 months. Although AZA was administered after relapse in our patient, we expected an epigenetic and GVL effect. Our patient may have showed CR because we administered AZA when there were few leukemic cells such as the cytogenetic relapse. Platzbecker et al reported that pre‐emptive therapy with AZA can prevent or substantially delay hematological relapse in measurable residual disease (MRD)‐positive patients with MDS or AML who are at high risk of relapse.^18^ Therefore, it may be clinically meaningful to administer AZA as maintenance therapy immediately after allo‐HSCT for AML with *FUS‐ERG*.

He developed recurrence in the BM and CNS during AZA therapy. Thus, concurrent treatment for recurrence in the BM and CNS was necessary, and we suggested the use of the TIT during administration of AZA.

In conclusion, expression of the CD56 antigen in AML with *FUS‐ERG* is associated with a poor prognosis, and achieving CR with allo‐HSCT is difficult. A novel therapeutic approach is needed for this clinical condition, and we suggest epigenetic therapy such as AZA as maintenance therapy after allo‐HSCT for AML with *FUS‐ERG*.

## CONFLICT OF INTEREST

All authors declare no conflicts of interest relevant to this article.

## AUTHOR CONTRIBUTIONS

Dai Keino: conceptualized and designed the study, drafted the initial manuscript, and approved the final manuscript. Takashi Mori, Mizuho Morimoto, Kensuke Kondo, and Tetsuya Mori: performed the initial analyses, and reviewed and revised the manuscript. Akitoshi Kinoshita: designed the data collection instruments, coordinated and supervised data collection, and critically reviewed the manuscript. All authors approved the final manuscript and agreed to be accountable for all aspects of the work.
